# Supply chain transparency and the availability of essential medicines

**DOI:** 10.2471/BLT.20.267724

**Published:** 2021-01-21

**Authors:** Christine Årdal, Enrico Baraldi, Peter Beyer, Yohann Lacotte, DG Joakim Larsson, Marie-Cécile Ploy, John-Arne Røttingen, Ingrid Smith

**Affiliations:** a Antimicrobial Resistance Centre, Norwegian Institute of Public Health, Postboks 222 Skøyen, 0213, Oslo, Norway.; b Department of Civil and Industrial Engineering, Uppsala University, Uppsala, Sweden.; c Department of Global Coordination and Partnership, World Health Organization, Geneva, Switzerland.; d RESINFIT, University of Limoges, Limoges, France.; e Department of Infectious Diseases, University of Gothenburg, Gothenburg, Sweden.; f Research Council of Norway, Lysaker, Norway.; g Department of Research and Development, Haukeland University Hospital, Bergen, Norway.

Sustainable access to essential medicines is crucial at all times, especially during a pandemic when health-care systems are operating at maximum capacity and there is an increased demand for life-saving supplies. Moreover, in pandemics, not only health-care systems but also global medicine supply chains are under severe stress. Shortages of medicines, which were common before 2020,^1^^,^^2^ have been exacerbated by the coronavirus disease 2019 (COVID-19) pandemic because of increased demand, lockdowns, border closures and hoarding.^3^^,^^4^ The supply of medicines could be improved by increasing the transparency of the complicated and fractured supply chain, starting upstream at the sources of active pharmaceutical ingredients.

Production of the active pharmaceutical ingredients that form the basis of every medicine is highly concentrated in only a few countries. China is the world’s largest producer, with an estimated 40% share of global production.^5^ India, the world’s largest provider of generic medicines, procures almost 70% of its active pharmaceutical ingredients from China.^6^ Yet the exact number and geographical distribution of producers remain elusive because companies that market medicines do not publish details of the sources of their active ingredients. Producers of the active pharmaceutical ingredients of a specific medicine are known only to the marketing authorization holder and the regulatory authority – neither buyers nor the public have access to this information. Thus, the fact that several companies may be selling a specific medicine in a particular country does not mean that there is a truly competitive market in that country capable of providing an ample supply. Prominent examples of global supply failures include: (i) a fire in 2017 at a factory in China producing active pharmaceutical ingredients that resulted in a global shortage of the antibiotic combination piperacillin–tazobactam;^1^ and (ii) insufficient production capacity of the sedative propofol to meet demand during the COVID-19 pandemic,^3^ which led some countries to reserve veterinary propofol for human use.^7^ However, supply failures of active pharmaceutical ingredients are common and affect the provision of medicines in all countries.^2^^,^^3^^,^^8^

Faced with more frequent shortages of medicines, many countries have acted to improve the management of supply chain interruptions, such as establishing a public register of shortages, but they have not yet increased the transparency of the supply chain. Marketing authorization holders are often contractually obligated to notify procurers when they are unable to supply a medicine and, in some cases, they are also obligated to bear the costs of replacement medicines.^2^ A recent study found that 19 countries (mostly in Europe) required marketing authorization holders to report anticipated shortages between 5 days and 6 months in advance, with the most common notice period being 2 months.^9^ Although these time frames may be sufficient for procurers to react to impending shortages, they will be insufficient to avoid shortages caused by the failure of the sole producer of an active pharmaceutical ingredient.

With greater supply chain transparency, governments would be able to work more proactively and collectively to identify limiting factors in the supply chain and, thereby, avoid shortages. Each national regulatory authority knows which active pharmaceutical ingredient producers are affiliated with each of the medicines marketed in their country. However, regulators cannot share this information with the public or government or even with regulators in other countries as the information is considered confidential. This situation leaves regulators in a quandary: they may not know if it is only medicine suppliers in their country who rely on a few producers or on a specific geographical region or if this is indeed the case for all suppliers of a particular medicine. To anticipate and avoid medicine shortages, countries need to understand the true nature of global supply chains so they can design effective mitigation measures for each medicine. Otherwise countries may be persuaded to intervene in the medicine supply without fully understanding the cost–effectiveness of a particular intervention. For example, many countries are currently discussing the local production of critical medicines (e.g. antibiotics) to meet their own needs.^4^ Yet cheaper and more efficient alternatives may be available, such as providing incentives for the geographical diversification of suppliers (including producers of active pharmaceutical ingredients), which would ultimately benefit all countries.

Private companies prefer their manufacturing and distribution practices to be kept secret for several reasons. For example, transparency would give competitors an insight into supply chains and could reveal supply weaknesses or financial details: an exact knowledge of the factories involved allows costs to be calculated fairly precisely. In addition, as many national and hospital medicine procurement agencies still tender almost solely on the basis of price,^2^ transparency may enable larger manufacturers to utilize financial information to drive out competitors. However, procurers are starting to value the benefits of a predictable supply and are applying the principle of multiple sourcing (i.e. they have several providers for each medicine where possible).^10^ Ideally, during the selection process, procurers should not only base their appraisals on price but should also consider whether supply chains are independent, resilient and meet environmental standards – characteristics that would give a company a competitive advantage in tendering and price negotiations.

Regulatory agencies should publish the source of the active pharmaceutical ingredients for each registered medicine along with the usual information. Through the efforts of the European Union’s Joint Action on Antimicrobial Resistance and Healthcare-Associated Infections and in light of supply challenges related to COVID-19, some European countries are considering moving towards greater supply chain transparency. As an example of how this can be done, the New Zealand Medicines and Medical Devices Safety Authority provides publicly available information on the names and locations of: (i) active pharmaceutical ingredient producers; (ii) finished product manufacturers; (iii) product sponsors; and (iv) the marketers of products.^11^ Recent reports have called for similar actions in the United States of America, including active monitoring of medicine supplies and increased supply chain transparency.^3^^,^^10^

In addition to enabling countries to better anticipate shortages and avoid them, supply chain transparency has other collective advantages. The discharge of wastewater during the manufacture of drugs can promote the development and spread of antimicrobial resistance and cause serious local environmental pollution that may also have public health implications.^12^ Greater transparency about the supply chain would increase pressure on international companies to monitor their sources of active pharmaceutical ingredients and enable engaged citizens to put pressure on governments and hospitals to ensure that any medicines they procure have been produced in a way that respects relevant environmental standards.

The practice of keeping medicine supply chains secret conflicts with public health needs. Without accurate information, procurers cannot proactively develop cost-effective plans for ensuring the sustainable, continuous supply of essential medicines. At the same time, procurers must ensure that suppliers are rewarded for maintaining robust supply chains and meeting environmental standards. Greater transparency is an essential first step in improving the medicines supply chain in a way that will benefit public health.
